# EXPLORING FALL RISK MANAGEMENT OF OLDER ADULTS IN A SAMPLE OF RURAL HEALTH CLINICS

**DOI:** 10.1093/geroni/igae098.3972

**Published:** 2024-12-31

**Authors:** Dawn Venema, Yao Yao, Victoria Kennel

**Affiliations:** College of Allied Health Professions, University of Nebraska Medical Center, Omaha, Nebraska, United States; College of Allied Health Professions, University of Nebraska Medical Center, Omaha, Nebraska, United States; College of Allied Health Professions, University of Nebraska Medical Center, Omaha, Nebraska, United States

## Abstract

Fall risk management (FRM) is an important component of primary care for older adults. Rural populations are aging more rapidly than urban ones, increasing the importance of FRM in rural communities. The Centers for Disease Control’s (CDC) Stopping Elderly Accidents, Deaths, and Injuries (STEADI) toolkit supports FRM implementation, including fall risk screening, assessing, and intervening. However, factors such as gaps in knowledge, time and financial constraints, and lack of patient engagement can limit FRM implementation. Using questions based on the STEADI toolkit, we surveyed Nebraska Rural Health Clinics (RHCs) to explore current FRM practices, facilitators, and barriers; 27% responded. Eighty-two percent screened older adult patients for fall risk. The percent of responding RHCs that conducted the following assessments for over half of their patients identified at-risk were: home environment (70%), comorbidities (45%), gait/strength/balance (40%), vitamin D (34%), orthostatic hypotension (21%), medication (17%), and footwear (17%). External reporting of quality measures focused on screening, assessment, and care plan documentation. Long-term FRM impacts (fall-related emergency department visits, hospitalizations) were rarely tracked, nor required for external reporting. Facilitators of FRM were availability of rehabilitation therapists in the community, time to conduct screening and assessments, and the Medicare Wellness Visit. Barriers were time to provide interventions, reimbursement, and patient receptivity to FRM activities. We are developing a quality improvement program called Coordinated Action Toward Community Health: RedUce Risk And Limit (CATCH RURAL) Falls to facilitate implementation of the CDC’s STEADI toolkit in RHCs for older adults including possible adaptations for FRM in rural communities.

